# Allele and Haplotype Frequencies of HLA-A, -B, -C, and -DRB1 Genes in 3,750 Cord Blood Units From a Kinh Vietnamese Population

**DOI:** 10.3389/fimmu.2022.875283

**Published:** 2022-06-29

**Authors:** Tran Ngoc Que, Nguyen Ba Khanh, Bach Quoc Khanh, Chu Van Son, Nguyen Thi Van Anh, Tran Thi Thuy Anh, Pham Dinh Tung, Nguyen Dinh Thang

**Affiliations:** ^1^ Stem Cell Bank, National Institute of Hematology and Blood Transfusion, Pham Van Bach, Cau Giay, Hanoi, Vietnam; ^2^ Department of Hematology, Hanoi Medical University, 1 Ton That Tung, Dong Da, Hanoi, Vietnam; ^3^ Key Laboratory of Enzyme and Protein Technology, VNU University of Science, Vietnam National University-Hanoi, 334 Nguyen Trai, Thanh Xuan, Hanoi, Vietnam; ^4^ Faculty of Biology, VNU University of Science, Vietnam National University-Hanoi, 334 Nguyen Trai, Thanh Xuan, Hanoi, Vietnam; ^5^ Department of Probability and Statistics, Faculty of Mathematics–Mechanics–Informatics, VNU University of Science, Vietnam National University, 334 Nguyen Trai, Thanh Xuan, Hanoi, Vietnam

**Keywords:** human leukocyte antigen (HLA), allele, haplotype, cord blood, Kinh Vietnamese population

## Abstract

The frequencies and diversities of human leukocyte antigen (HLA) alleles and haplotypes are representative of ethnicities. Matching HLA alleles is essential for many clinical applications, including blood transfusion, stem cell transplantation, and tissue/organ transplantation. To date, the information about the frequencies and distributions of HLA alleles and haplotypes among the Kinh Vietnamese population is limited because of the small sample size. In this study, more than 3,750 cord blood units from individuals belonging to the Kinh Vietnamese population were genotyped using PCR sequence-specific oligonucleotide (PCR-SSO) for HLA testing. The results of the study demonstrated that the most frequently occurring HLA-A, -B, -C, and -DRB1 alleles were A*11:01 (25%), A*24:02 (12.3%), A*02:01 (11.2); A*03:03 (8.95%), A*02:03 (7.81%), A*29:01 (7.03%); B*15:02 (15.1%), B*46:01 (10.7%), B*58:01 (7.65%), B*38:02 (7.29%); C*08:01 (17.2), C*07:02 (16.2%), C*01:02 (15.2), C*03:02 (8.3%), C*15:05 (6.13); DRB1*12:02 (31.0%), DRB1*09:01 (10.47%), DRB1*15:02 (7.54%); DRB1*07:01 (6.68%), DRB1*10:01 (6.63%), respectively, with the highest allele diversity level observed in locus B (93 alleles). The most frequent haplotypes of two-locus combinations of HLA-A–B, HLA-A–C, HLA-A–DRB1, HLA-B–C, HLA-B–DRB1, and HLA-C–DRB1 haplotypes were A*11:01–B*15:02 (7.63%), A*11:01–C*08:01 (7.98%), A*11:01–DRB1*12:02 (10.56%), B*15:02–C*08:01 (14.0%), B*15:02–DRB1*12:02 (10.47%), and C*08:01–DRB1*12:02 (11.38%), respectively. In addition, the most frequent haplotypes of three- and four-locus sets of HLA-A–B–C, HLA-A–B–DRB1, HLA-A–C–DRB1, HLA-B–C–DRB1, and HLA-A–B–C–DRB1 were A*11:01–B*15:02–C*08:01 (7.57%), A*11:01–B*15:02–DRB1*12:02 (5.39%), A*11:01–C*08:01–DRB1*12:02 (5.54%), B*15:02–C*08:01–DRB1*12:02 (10.21%), and A*11:01–B*15:02–C*08:01–DRB1*12:02 (5.45%), respectively. This study provides critical information on the frequencies and distributions of HLA alleles and haplotypes in the Kinh Vietnamese population, accounting for more than 85% of Vietnamese citizens. It paves the way to establish an umbilical cord blood bank for cord blood transplantation programs in Vietnam.

## Introduction

Haematopoietic stem cell transplantation is an effective treatment option for various blood diseases. Donors and recipients with blood relations are most suited for stem cell engraftment because of their high compatibility, ensuring the optimal grafting effect (1, 2). However, in many cases, patients do not have close relationships with the donors. Therefore, the patients could not have donors with high compatibility with the human leukocyte antigen (HLA) as finding a donor with suitable HLA for grafting is challenging. Consequently, cord blood banks have been developed (3–6). Cord blood, which is readily available and easy to collect, has an extremely low risk for donors and has been used in numerous clinics (5–8).

The HLA system includes two classes: HLA-A, -B, and -C, which correspond to major histocompatibility complex (MHC) class I, and HLA-DRB1, -DQA1, -DQB1, and -DPB1, which correspond to MHC class II. MHC class I is present on the surface of all cell types and is recognised byT-CD8^+^ cells (9, 10). Incompatibility in genotypes of both HLA classes I and II between the donor and recipient causes organ rejection and graft failure in haematopoietic stem cell transplantation (9). The primary requirement for cord blood grafting is HLA compatibility of at least four of the six loci from MHC classes I and II (10, 11).

Cord blood was previously regarded as clinical waste; however, it has recently been recognised as a promising and effective source for treating various diseases, particularly haematopoietic disorder-related diseases (5–8). When applying stem cells for transplantation, finding compatible donors among the relations is problematic. Therefore, cord blood samples stored in blood banks are an excellent alternative. Graft rejection is a serious complication that may occur after transplantation. Therefore, storing cord blood is beneficial for children and their relatives in the future. Because of its abundant sources, using cord blood in clinics will help reduce the treatment cost and time (3–6). Cord blood stem cells can differentiate into various cells, including red blood cells, white blood cells, and platelets. Thus, cord blood has become very promising for clinical applications in treating multiple aggressive pathologies, such as anaemia, lymphoma, and leukaemia (1, 5–8, 12).

Therefore, we investigated and reported information on the frequency, diversity, and distribution of alleles and haplotypes in four HLA loci, including HLA-A, HLA-B, HLA-C, and HLA-DRB1, in 3,750 cord blood units of the Kinh Vietnamese population, to establish an umbilical cord blood bank at the National Institute of Haematology and Blood Transfusion of Vietnam.

## Materials And Methods

### Statistical Analysis

Population statistical analyses and diversity of HLA genotypes were calculated using Arlequin 3.5 software. In particular, the individual allele frequencies of each locus and the common haplotype frequencies of two, three, and four loci were determined using the expectation–maximisation (EM) algorithm. An exact test using a Markov chain algorithm was performed to test for deviation from Hardy–Weinberg equilibrium (13, 14).

### Population and Sample Collection

A cross-sectional study was conducted on a cohort of 3,776 Kinh Vietnamese healthy pregnant women for cord blood samples. The samples were collected between May 2014 and December 2019. Cord blood donors mostly came from every province in northern and north-central Vietnam. Cord blood samples of ≥80 mL in volume, without blood cluster or bizarre colours, collected within 24 h of birth, mean corpuscular volume (MCV) of ≥95 fl, no abnormal haemoglobin confirmed by high-performance liquid chromatography (HPLC) analysis, number of nucleated cells ≥10^9^cells/mL, negative for infectious pathogens including hepatitis C virus (HCV), hepatitis B virus (HBV), human immunodeficiency virus (HIV), cytomegalovirus (CMV), and bacteria were collected and stored at −196°C in liquid nitrogen tanks at the Umbilical Cord Blood Bank, National Institute of Haematology and Blood Transfusion, Vietnam.

### HLA Genotyping

All cord blood samples were used for DNA extraction using the QIAamp DNA Blood Mini Kit (Qiagen, Hilden, Germany), from which 40–120 ng of genomic DNA was required for each locus typing. Medium-to-high resolution (second field) HLA typing (MHR-2F) of four loci, including A, B, C, and DRB1, was conducted by PCR sequence-specific oligonucleotide (PCR-SSO) using the LIFECODES HLA SSO kit (Luminex, Austin, TX, USA) on a Luminex 200 system, and analysed with Xponent 3.1 software connected to the IMGT (International ImMunoGeneTics) database version 3.43 library to provide us with the well-documented allele (WDA) (15–17). Blind external controls supplied by UK External Quality Assessment Services (UK EQAS) were used to confirm the accuracy of the four HLA loci identified. The procedure has been standardized under ISO15189:2012 criteria and is routinely used at Stem Cell Bank, Vietnam National Institute of Haematology and Blood Transfusion. Heterogeneous or homogeneous forms of the HLA gene were found. HLA typing was performed from June 2015 to December 2020. A few samples with ambiguous results in the 4-digit assignment were excluded from further investigation in this study. HLA typing was performed from June 2015 to December 2020.

## Results

### Cohort Haematological Characteristics

In this study, 3,776 cord blood samples were collected. The proportion of ambiguous results in the 4-digit assignment (“medium-to-high” resolution) was 0.68% (26/3,776) among the total 3,776 cord blood units. These 26 samples with ambiguous data were removed, and the remaining 3,750 samples were included in this research for further investigation. The characteristics of the study cohort are shown in **Table 1**. Among 3,750 donors, there were 1,976 men (52.7%) and 1,774 women (47.3%). The distribution of blood types, including A, B, AB, and O, was almost the same in men and women. The percentage of donors with O-type was the highest at 45.5%, the AB-type was the lowest at 4.8%, and the A-type and B-type contributed to 20.9% and 28.8%, respectively. In particular, the number of donors with the O-type was 2.2-, 1.6-, and 9.5-fold higher than the A-, B-, and AB-types, respectively (**Table 1**). This report agrees with previous reports on Kinh Vietnamese population (15, 18).

**Table 1:** Haematological characteristics of the cohort.

Blood type				
Sex	A	B	AB	O
**Male**	397 (10.6%)	575 (15.3)	89 (2.4%)	915 (24.4%)
**Female**	388 (10.3)	504 (13.4)	91 (2.4%)	791 (21.1%)
**Total**	785 (20.9%)	1079 (28.8)	180 (4.8%)	1,706 (45.5%)

### Individual Allele Frequencies

**Figure 1:**
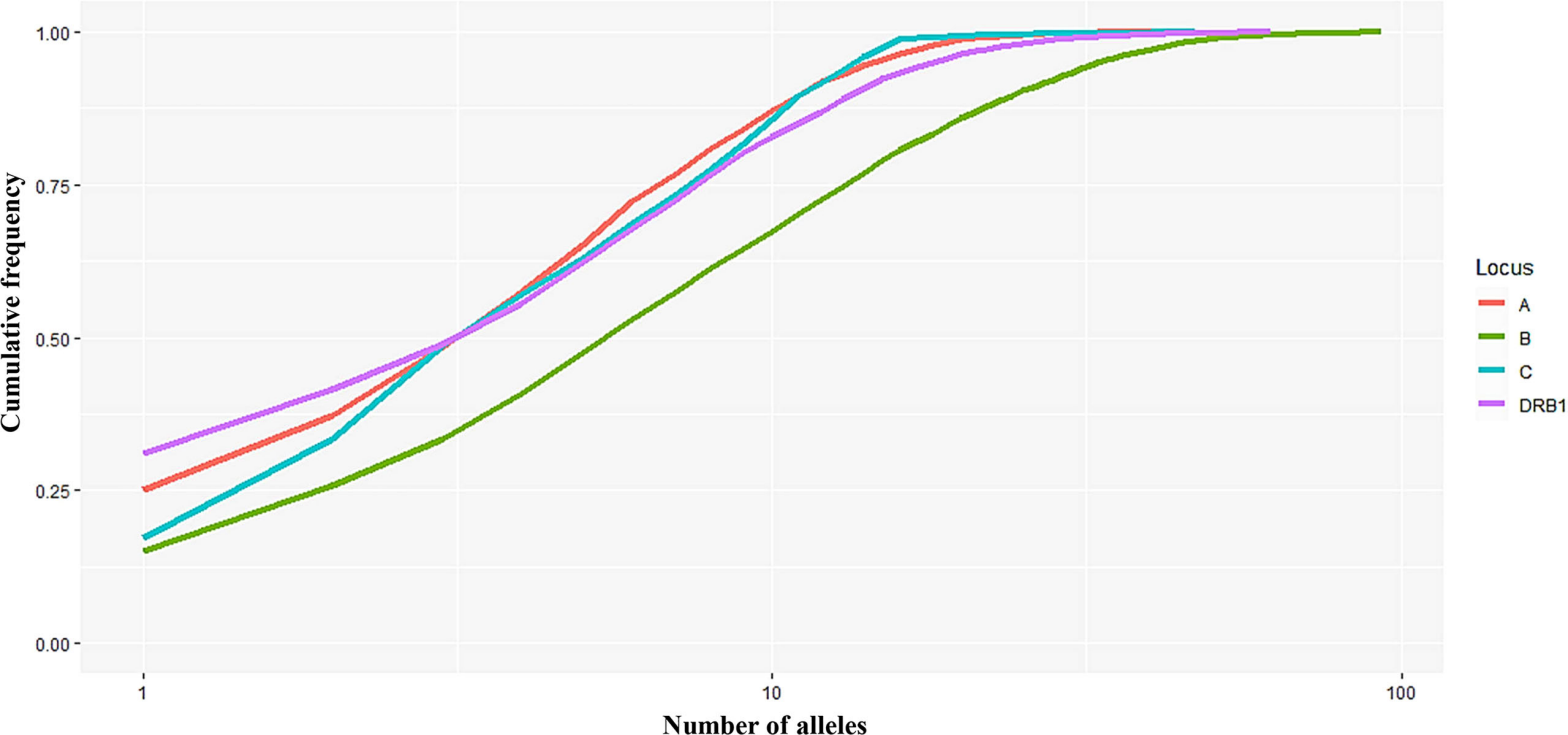
Curves showing the cumulative frequency of alleles for each HLA locus (A, B, C, and DRB1). The number of alleles (*n*) on the *x*-axis is expressed on a logarithmic scale.

**Figure 2:**
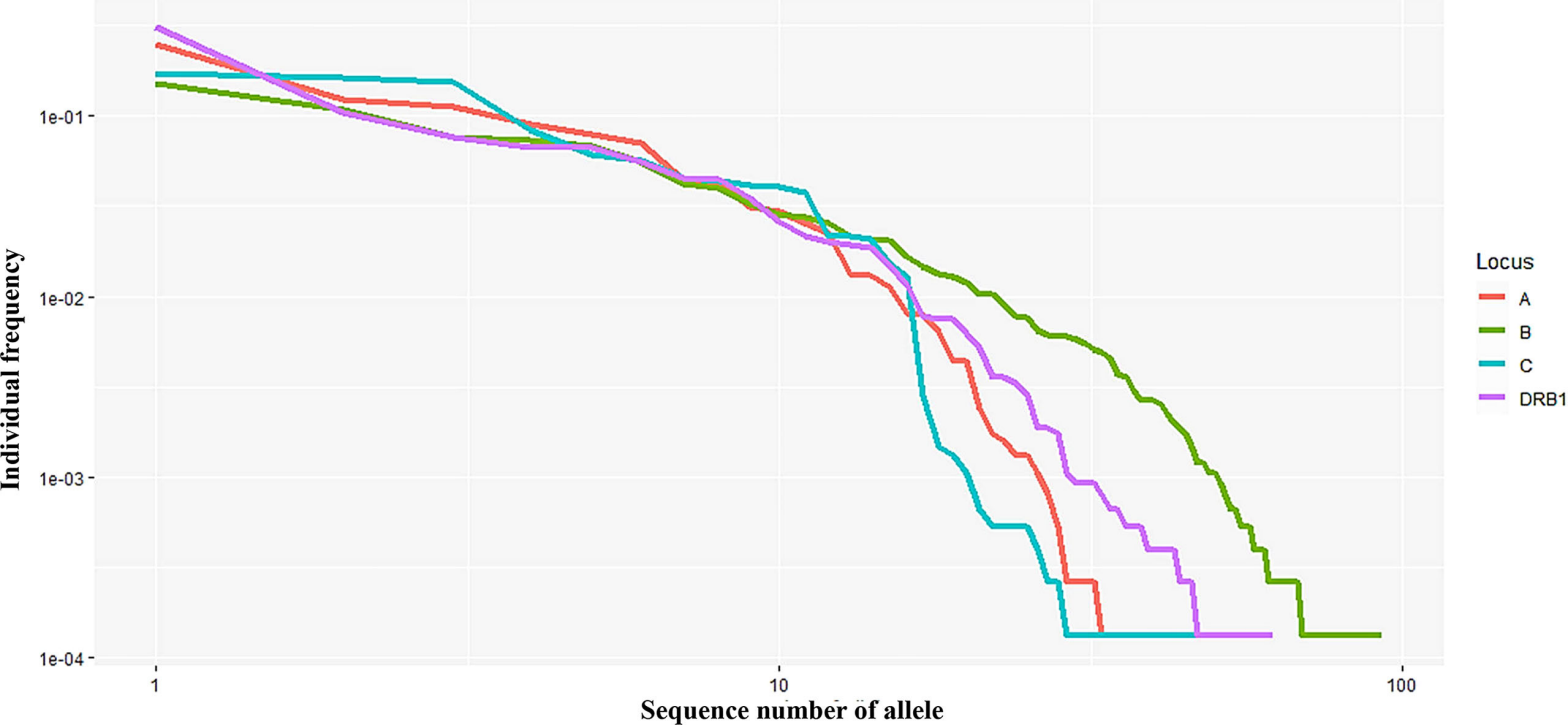
Curves showing the allele frequency of each HLA locus (A, B, C, and DRB1) in descending order.Both the *x*- and *y*-axes are expressed on a logarithmic scale.

In this study, 40, 93, 47, and 62 individual alleles of HLA-A, -B, -C, and -DRB1, respectively, were found in the Kinh Vietnamese cohort (**Table 2**). This result indicated that locus B exhibited the highest polymorphism, with almost double the number of alleles than loci A, C, or DRB1. In the locus A (HLA-A), the most frequent alleles were A*11:01 (25%), A*24:02 (12.3%), A*02:01 (11.2), A*03:03 (8.95%), A*02:03 (7.81%), and A*29:01 (7.03%). In the locus B (HLA-B), the most frequent alleles were B*15:02 (15.1%), B*46:01 (10.7%), B*58:01 (7.65%), B*38:02 (7.29%), and B*15:25 (5.55%). In the locus C (HLA-C), the most frequent alleles were C*08:01 (17.2), C*07:02 (16.2%), C*01:02 (15.2), C*03:02 (8.3%), C*15:05 (6.13), and C*03:04 (5.61%). And, in the locus DRB1 (HLA-DRB1), the most frequent were DRB1*12:02 (31.0%), DRB1*09:01 (10.47%), DRB1*15:02 (7.54%); DRB1*07:01 (6.68%), DRB1*10:01 (6.63%), and DRB1*03:01 (5.53%). Of the 40 alleles at locus A, there were six alleles with frequencies of at least 5%, accounting for 72.3%, 15 alleles with frequencies of over 1%, accounting for 95.6, and 13 rare alleles with frequencies below 0.1%, accounting for 0.35%. Of the 93 alleles in locus B, only six alleles with frequencies of over 5% accounted for 53.1%, 21 alleles with frequencies greater than 1% accounted for 87.9%, and 42 rare alleles with frequencies below 0.1% accounted for 1.13%. In 47 alleles at locus C, there were six alleles with frequencies of over 5%, accounting for 68.7%, 16 alleles with frequencies of over 1%, accounting for 98.7%, and 26 rare alleles with frequencies below 0.1%, accounting for 0.63%. Among the 62 alleles of HLA-DRB1, there were six alleles with frequencies of over 5%, accounting for 67.8%, 16 alleles with frequencies greater than 1%, accounting for 93.4%, and 32 rare alleles with frequencies below 0.1%, accounting for 1.14% (**Table 2**). The allele HLA-DRB1*12:02 was the most common individual HLA allele present in four loci: HLA-A, -B, -C, and -DRB1, with a frequency of 31%. The Hardy–Weinberg equilibrium analysis results are presented in **Table 3**. The observed heterozygosity in all four loci of HLA-A, HLA-B, HLA-C, and HLA-DRB1 was significantly lower than expected by chance in this large cohort. With a small *P* value (<0.01) for each locus in this cohort, the observed heterozygosity was significantly less than expected. This suggests that the loci deviated from the Hardy–Weinberg equilibrium. An overview of the cumulative frequency at 50%, 75%, 90%, 95%, and 100% of the individual alleles of all four loci HLA-A, HLA-B, HLA-C, and HLA-DRB1 is presented in **Figure 1**, and their distributions are shown in **Figure 2** (see also **Supplemental Material 1**). The polymorphic levels of HLA-A and HLA-C were almost the same and much lower than HLA-B, especially because of the number of frequent alleles (40 and 47 *vs* 93). The polymorphic HLA-DRB1 (62) was higher than HLA-A and HLA-C but lower than HLA-B. Other studies have also reported that HLA-A and HLA-C have a very similar amount of information, and HLA-B is the most polymorphic locus in not only three loci of HLA class I but in all six HLA loci for both HLA class I and HLA class II (19–23). Although the number of high-frequency (occurring frequency >5%) alleles in HLA-A, -B, -C, and -DRB1 were the same (six alleles for each), the number of rare alleles (occurring frequency <0.1%) in HLA-B was approximately 3-, 1.6-, and 1.3-fold higher than those in HLA-A, -C, and -DRB1, respectively. In addition, a test of linkage disequilibrium for all pairs of four loci (HLA-A, -B, -C, and -DRB1) was conducted, and the results are shown in **Table 4**. Significant linkage disequilibrium was observed for all pairs of the four loci (HLA-A, -B, -C, and -DRB1).

**Table 2:** HLA-A, -B, -C, and -DRB1 allele frequencies in cord blood of the Kinh Vietnamese donors.

HLA-A	Freq.					HLA-DRB1	Freq.
A*11:01	0.249601	B*40:06	0.010378	B*40:63	0.000133	DRB1*12:02	0.309606
A*24:02	0.122805	B*18:01	0.010245	B*44:07	0.000133	DRB1*09:01	0.104710
A*02:01	0.112161	B*27:06	0.009047	B*46:06	0.000133	DRB1*15:02	0.075439
A*33:03	0.089542	B*13:02	0.007850	B*49:01	0.000133	DRB1*07:01	0.066791
A*02:03	0.078100	B*52:01	0.007717	B*51:03	0.000133	DRB1*10:01	0.066259
A*29:01	0.070250	B*35:01	0.006519	B*51:04	0.000133	DRB1*03:01	0.055349
A*33:01	0.044172	B*56:01	0.006120	B*05:12	0.000133	DRB1*08:03	0.044971
A*02:06	0.042709	B*37:01	0.005987	B*54:26	0.000133	DRB1*04:05	0.044039
A*11:02	0.031533	B*40:02	0.005987	B*56:10	0.000133	DRB1*15:01	0.034992
A*01:01	0.029404	B*48:03	0.005854	B*58:03	0.000133	DRB1*14:01	0.026078
A*24:07	0.025546	B*35:03	0.005455	B*67:01	0.000133	DRB1*04:03	0.021687
A*26:01	0.022219	B*56:04	0.005056	B*81:01	0.000133	DRB1*13:12	0.020090
A*31:01	0.013305	B*48:01	0.004923	**HLA-C**	**Freq.**	DRB1*16:02	0.019159
A*24:03	0.013172	B*18:02	0.004524	C*08:01	0.172432	DRB1*13:02	0.018627
A*30:01	0.011309	B*15:18	0.003725	C*07:02	0.162054	DRB1*11:01	0.015035
A*03:01	0.008116	B*56:02	0.003592	C*01:02	0.152076	DRB1*14:04	0.011575
A*34:01	0.007983	B*08:01	0.003060	C*03:02	0.083023	DRB1*14:05	0.007850
A*24:10	0.006386	B*15:11	0.002661	C*15:05	0.061336	DRB1*11:06	0.007584
A*11:04	0.004391	B*15:13	0.002661	C*03:04	0.056147	DRB1*13:01	0.007451
A*68:01	0.004391	B*15:27	0.002661	C*04:03	0.044838	DRB1*04:06	0.006253
A*74:01	0.002395	B*15:21	0.002528	C*04:01	0.043773	DRB1*12:01	0.005189
A*74:05	0.001730	B*50:01	0.002262	C*06:02	0.040979	DRB1*11:04	0.003592
A*32:01	0.001597	B*39:05	0.001996	C*03:03	0.04058	DRB1*12:07	0.003592
A*02:11	0.001330	B*46:02	0.001863	C*07:01	0.037786	DRB1*04:04	0.003326
A*29:02	0.001330	B*73:01	0.001730	C*12:02	0.021687	DRB1*01:01	0.002794
A*02:05	0.001064	B*38:01	0.001464	C*14:02	0.021687	DRB1*01:02	0.001863
A*03:02	0.000798	B*27:05	0.001197	C*15:02	0.020756	DRB1*04:01	0.001863
A*02:02	0.000532	B*55:01	0.001197	C*12:03	0.015168	DRB1*04:10	0.001730
A*11:03	0.000266	B*39:09	0.001064	C*07:04	0.012773	DRB1*16:12	0.001064
A*11:12	0.000266	B*51:06	0.001064	C*04:06	0.002794	DRB1*14:18	0.000931
A*23:01	0.000266	B*39:15	0.000931	C*02:02	0.001464	DRB1*16:01	0.000931
A*03:07	0.000266	B*41:01	0.000798	C*08:03	0.001330	DRB1*08:02	0.000931
A*01:03	0.000133	B*15:10	0.000665	C*16:02	0.001064	DRB1*14:07	0.000798
A*02:38	0.000133	B*15:17	0.000665	C*01:03	0.000665	DRB1*04:07	0.000665
A*24:01	0.000133	B*15:15	0.000532	C*15:09	0.000532	DRB1*08:01	0.000665
A*24:05	0.000133	B*27:03	0.000532	C*03:01	0.000532	DRB1*12:16	0.000532
A*24:17	0.000133	B*44:02	0.000532	C*03:09	0.000532	DRB1*14:54	0.000532
A*24:43	0.000133	B*27:07	0.000399	C*05:01	0.000532	DRB1*07:03	0.000532
A*29:03	0.000133	B*39:03	0.000399	C*17:01	0.000399	DRB1*13:07	0.000399
A*68:02	0.000133	B*58:02	0.000399	C*15:25	0.000266	DRB1*14:02	0.000399
**HLA-B**	**Freq.**	B*15:35	0.000266	C*06:42	0.000266	DRB1*14:10	0.000399
B*15:02	0.151144	B*15:46	0.000266	C*01:01	0.000133	DRB1*16:10	0.000399
B*46:01	0.107105	B*39:06	0.000266	C*01:05	0.000133	DRB1*08:04	0.000399
B*58:01	0.076503	B*40:09	0.000266	C*01:06	0.000133	DRB1*15:35	0.000266
B*38:02	0.072911	B*51:07	0.000266	C*01:17	0.000133	DRB1*04:08	0.000266
B*07:05	0.067855	B*56:03	0.000266	C*12:14	0.000133	DRB1*09:06	0.000266
B*15:25	0.055482	B*58:16	0.000266	C*14:03	0.000133	DRB1*10:02	0.000133
B*13:01	0.041644	B*07:01	0.000266	C*15:03	0.000133	DRB1*11:46	0.000133
B*40:01	0.039782	B*13:07	0.000133	C*15:04	0.000133	DRB1*12:03	0.000133
B*44:03	0.032597	B*15:03	0.000133	C*15:12	0.000133	DRB1*12:12	0.000133
B*35:05	0.028473	B*15:05	0.000133	C*15:46	0.000133	DRB1*13:58	0.000133
B*57:01	0.027275	B*15:07	0.000133	C*16:01	0.000133	DRB1*13:60	0.000133
B*54:01	0.025679	B*15:08	0.000133	C*03:08	0.000133	DRB1*14:03	0.000133
B*51:01	0.021554	B*16:41	0.000133	C*04:02	0.000133	DRB1*14:28	0.000133
B*15:12	0.020623	B*18:08	0.000133	C*04:04	0.000133	DRB1*15:03	0.000133
B*55:02	0.020357	B*25:05	0.000133	C*06:03	0.000133	DRB1*15:06	0.000133
B*15:01	0.016631	B*35:02	0.000133	C*06:08	0.000133	DRB1*03:02	0.000133
B*07:02	0.014502	B*35:11	0.000133	C*07:26	0.000133	DRB1*03:05	0.000133
B*51:02	0.013438	B*35:32	0.000133	C*07:29	0.000133	DRB1*03:07	0.000133
B*39:01	0.012906	B*35:58	0.000133	C*07:56	0.000133	DRB1*03:23	0.000133
B*27:04	0.011841	B*40:27	0.000133			DRB1*07:05	0.000133
						DRB1*08:09	0.000133

Bold values in the Table 2 are to separate different loci for ease following.

**Table 3:** Hardy–Weinberg equilibrium with an exact test using a Markov chain.

Locus	Genotype	Observed heterozygosity	Expected heterozygosity	*P* value	S.D.
**A**	3758	0.87786	0.88362	0.00000	0.00000
**B**	3758	0.93241	0.93729	0.01608	0.00004
**C**	3758	0.88744	0.89673	0.00068	0.00001
**DRB1**	3758	0.85977	0.86757	0.00000	0.00000

**Table 4:** Linkage disequilibrium for all pairs ofthe four loci (+: significance level = 0.0500).

Locus	A	B	C	DRB1
A	*	+	+	+
B	+	*	+	+
C	+	+	*	+
DRB1	+	+	+	*

(Linkage disequilibrium test for the four loci [HLA-A, -B, -C, and -DRB1 correspond with 0, 1, 2, and 3): Pair(0,1) LnLHood LD: −33501.32038; LnLHood LE: −38575.47508; Exact P = 0.00000 ± 0.00000; Chi-square test value = 10148.30939 (P = 0.00000, 3588 d.f.); Pair(0,2) LnLHood LD: −29520.02695, LnLHood LE: −33505.12195; Exact P = 0.00000 ± 0.00000; Chi-square test value = 7970.18999 (P = 0.00000, 1794 d.f.); Pair(1,2) LnLHood LD: −25562.82326; LnLHood LE: −38684.15862; Exact P = 0.00000 ± 0.00000, Chi-square test value = 26242.67073 (P = 0.00000, 4232 d.f.); Pair(0,3) LnLHood LD: −31686.72178, LnLHood LE: −34053.13059; Exact P = 0.00000 ± 0.00000, Chi-square test value = 4732.81762 (P = 0.00000, 2379 d.f.); Pair(1,3) LnLHood LD: −33568.25664; LnLHood LE: −39232.16727; Exact P = 0.00000 ± 0.00000, Chi-square test value = 11327.82126 (P = 0.00000, 5612 d.f.); Pair(2,3) LnLHood LD: −30180.47304; LnLHood LE: −34161.81414; Exact P = 0.00000 ± 0.00000, Chi-square test value = 7962.68218 (P = 0.00000, 2806 d.f.). +, significance level = 0.0500).

“ * ” NO TEST (because of the same locus).

### Haplotype Frequencies

Haplotype frequencies of two, three, and four loci combinations (A–B, A–C, A–DRB1, B–C, B–DRB1, C–DRB1, A-B-C, A–B–DRB1, A–C–DRB1, B–C–DRB1, and A-B-C–DRB1) are shown in **Supplemental Material 2**. The combinations of alleles in the two loci created 300, 341, 439, 471, 592, and 695 haplotypes of B–C, A–C, C–DRB1, A–DRB1, A–B, and B–DRB1, respectively. In the combinations of three loci, 695, 976, 1,071, 1,633, and 1,743 haplotypes of A–B–C, B–C–DRB1, A–C–DRB1, A–B–DRB1, and A–B–DRB1, respectively, were found. A total of 2,087 haplotypes of four HLA loci (A-B-C–DRB1) were recorded. The number of haplotypes with a frequency of over 5% of A–B, A–C, B–C, A–DRB1, B–DRB1, and C–DRB1 was only 3, 4, 5, 2, 2, and 2, respectively. The number of haplotypes with a frequency of over 1% of A–B, A–C, B–C, A–DRB1, B–DRB1, and C–DRB1 was 19, 24, 20, 20, 18, and 19, respectively (see **Supplemental Material 2**).

The highest haplotype frequency of HLA-A–B was A*11:01–B*15:02 (7.63%), followed by A*02:01–B*46:01 (5.97%), and A*29:01–B07:05 (5.34%). The highest haplotype frequency of HLA-A–C was A*11:01–C*08:01 (7.98%), followed by A*02:01–C*01:02 (6.51%), A*29:01–C*15:05 (5.37%), and A*11:01–C*07:02 (5.05%). The highest haplotype frequency of HLA-B–C was B*15:02–C*08:01 (14.0%), followed by B*46:01–C*01:02 (10.23%), B*58:01–C*03:02 (7.49%), B*38:02–C*07:02 (7.18%), and B*07:05–C*15:05 (5.83%). The highest haplotype frequency of HLA-A–DRB1 was A*11:01–DRB1*12:02 (10.56%), followed by A*24:02–DRB1*12:02 (5.04%). The highest haplotype frequency of HLA-B–DRB1 was B*15:02–DRB1*12:02 (10.47%), followed by B*46:01–DRB1*09:01 (5.20%). The highest haplotype frequency of HLA-C–DRB1 was C*08:01–DRB1*12:02 (11.38%), followed by HLA-C*01:02–DRB1*09:01 (5.49%).

**Figure 3:**
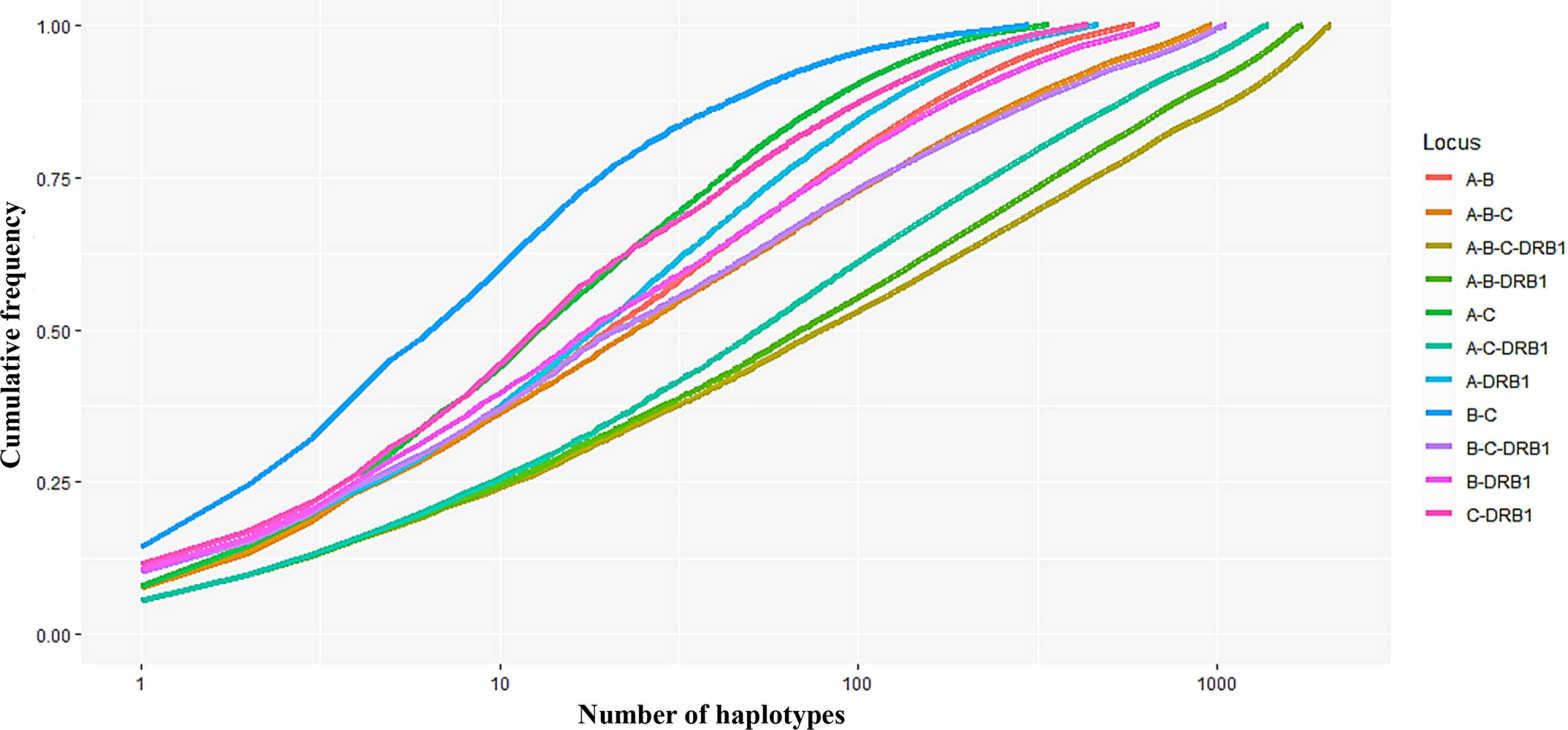
Curves showing the cumulative haplotype frequencies of each HLA locus-combination set (A–B, A–C, A–DRB1, B–C, B–DRB1, C–DRB1, A–B–C, A–B–DRB1, A–C–DRB1, B–C–DRB1, A–B–C–DRB1). The number of haplotypes (*n*) on the *x*-axis is expressed on a logarithmic scale.

**Figure 4:**
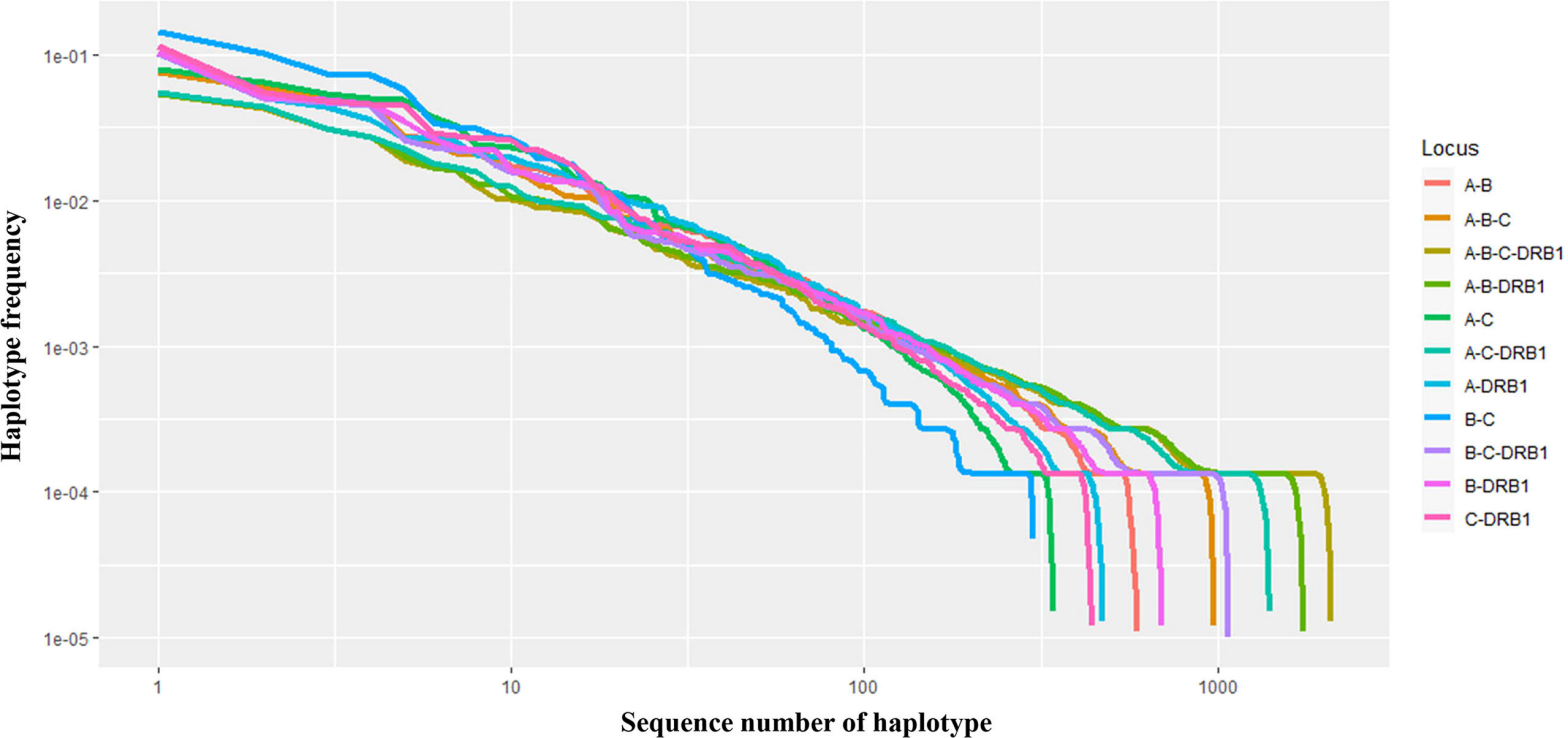
Curves showing the haplotype frequency of each set of loci (A–B, A–C, A–DRB1, B–C, B–DRB1, C–DRB1, A–B–C, A–B–DRB1, A–C–DRB1, B–C–DRB1, A–B–C–DRB1) in descending order. Both the *x*- and *y*-axes are expressed on a logarithmic scale.

In addition, the frequencies of haplotypes with combinations of three and four loci were also estimated and are shown in **Supplemental Material 2**. The highest haplotype frequency of HLA-A–B–C was HLA-A*11:01–B*15:02–C*08:01 (7.57%), followed by A*02:01–B*46:01–C*01:02 (5.89%), and A*29:01–B*07:05–C*15:05 (5.27%). The most frequent alleles of HLA-A–B–DRB1, HLA-A–C–DRB1, HLA-B–C–DRB1, and HLA-A–B–C–DRB1 were A*11:01–B*15:02–DRB1*12:02 (5.39%), A*11:01–C*08:01–DRB1*12:02 (5.54%), B*15:02–C*08:01–DRB1*12:02 (10.21%), and A*11:01–B*15:02–C*08:01–DRB1*12:02 (5.45%), respectively. In combinations of three or four loci, the haplotype distributions varied widely, with many haplotypes having frequencies below 0.1%. In particular, among 885 haplotypes of HLA-A–B–C, 1,743 haplotypes of HLA-A–B–DRB1, 1,633 haplotypes of HLA-A–C–DRB1, 1,071 haplotypes of HLA-B–C–DRB1, and 2,087 haplotypes of HLA-A–B–C–DRB1, 746 haplotypes (18.95%), 1,583 haplotypes (37.49%), 1,098 haplotypes (27.47%), 936 haplotypes (22.93%), and 1,939 haplotypes (41.53%), respectively, with rare frequencies of less than 0.1% (see **Supplemental Material 2).** The cumulative frequency is shown in **Figures 3**, **4**. The numbers of haplotypes covering the corresponding 50%, 75%, 90%, 95%, and 100% alleles are shown in **Table 5**. A total of 976, 1,633, 1,071, 1,743, and 2,087 haplotypes of HLA-A–B–C, HLA-A–B–DRB1, HLA-A–C–DRB1, HLA-B–C–DRB1, and HLA-A–B–C–DRB1, respectively, were recorded.

**Table 5:** Number of the most frequent haplotypes required to cover 50%, 75%, 90%, 95%, and 100% of the genes in each locus-combination set.

HLA loci combinations	Number of haplotypes required to cover				
	50%	75%	90%	95%	100%
**B-C**	7	19	55	94	300
**A-C**	13	42	98	149	341
**C-DRB1**	13	47	124	196	439
**A-DRB1**	19	48	144	218	471
**A-B**	21	79	196	297	592
**B-DRB1**	18	81	224	356	695
**A-B-C**	24	117	351	575	976
**B-C-DRB1**	22	115	393	672	1071
**A-C-DRB1**	54	259	837	1244	1633
**A-B-DRB1**	70	350	942	1317	1743
**A-B-C-DRB1**	79	455	1287	1663	2087

## Discussion

Transplantation rejection is a common issue. Six HLA loci correspond to MHC class I and MHC class II, with three loci of HLA-A, -B, and -C relating to class I and HLA-DRB1, -DQA1, -DQB1, and -DPB1 related to class II. The level of HLA compatibility required varies depending on the transplantation technique used. In organ or stem cell transplantation, the compatibility of five or even six loci between the donor and the recipient is necessary; however, compatibility of at least four of the six loci is acceptable in the case of cord blood transplantation. Approximately 500 cord blood banks have been established in 97 countries worldwide (3–5).

The Kinh people were the most predominant ethnic community in Vietnam and accounted for more than 85% of the Vietnamese population (15, 18). However, information on the frequency and distribution of HLA alleles in the Kinh Vietnamese cord blood is yet to be thoroughly investigated. Several studies have examined HLA alleles and haplotypes in the Kinh Vietnamese, but the sample size was quite limited, with only around 100 donors (14, 15). The National Institute of Haematology and Blood Transfusion in Vietnam is one of the largest and most important storage facilities for umbilical cord blood in Vietnam. For future transplantation use, HLA genotypes of every cord blood sample should be tested.

In this study, cord blood units from the Kinh Vietnamese cohort were collected and stored in the cord blood bank at the National Institute of Haematology and Blood Transfusion, and PCR-SSO with Luminex Technology, a routine method in the institute, was used for HLA genotyping of these samples. Actually, there are several studies recorded that Luminex Technology for HLA-typing may cause a few percentage of ambiguous results as the Reviewer 2 have mentioned. However, many laboratories have used this method to identify HLA genotypes at 4-digit assignment (described as “medium-to-high” resolution) with good results (21, 24). Typically, Park et al. conducted genotyping of HLA-A, HLA-B and HLA-DRB1 of more than ten thousand (10,918) Koreans by using Luminex Technology and they confirmed that this platform was possible for genotyping most of the samples at the level of 4-digit assignment, there were only few cases with ambiguous results that must be reconfirmed by Sequence-Based Typing (SBT) from Abbot Molecular, USA (21). In this study, because the procedure has been standardized under ISO15189:2012 criteria and is routinely used at Stem Cell Bank, Vietnam National Institute of Haematology and Blood Transfusion, the proportion of ambiguous results in the 4-digit assignment (“medium-to-high” resolution) was very low, only 0.68% (26/3,776) among the total 3,776 cord blood units. As we concentrated on big data analysiswith a large sample size, therefore, for convenience, we did not include these 26 samples in this report, and the remaining 3,750 samples were analysed.

The findings in this study regarding frequencies and distributions of alleles and haplotypes in the Kinh Vietnamese cohort were similar to those in previous reports (15, 18), but there were a few differences. Hoa et al. (15) and Do et al. (18) found that the three most frequent alleles for HLA-A were A∗11:01, A∗24:02, and A∗33:03, with frequencies of 22.77%, 12.87%, 10.89%, and 22.9%, and 13.8% and 11.5%, respectively; however, in this study, the three most frequent alleles in HLA-A were A*11:01, A*24:02, and A*02:01 with frequencies of 25%, 12.3%, and 11.2%, respectively. This indicated that in the small sample size, A*02:01 was not listed as a dominant allele with a frequency of less than 5%, but in the larger sample size, A*02:01 was listed in the top three among the most frequent alleles. Similarly, regarding the allele frequencies at locus B, although the B∗40:01 allele was classified as a dominant allele with a frequency of more than 6% in Hoa et al. (6.2%) (15) and Do et al. (7.92%) (18), it only occurred with a frequency of 3.98% in this study. Furthermore, although the three most frequent alleles at locus C were the same in the studies by Hoa et al. (15), Do et al. (18), and the current study, the ranked positions were not. In particular, C∗07:02 (21.8%), C∗01:02 (13.4%), and C∗08:01 (12.9%) were listed as dominant HLA-C alleles in Do et al. (15); C*01:02 (16.5%), C*08:01 (15.6%), and C*07:02 (14.7%) in Hoa et al. (15); and C*08:01 (17.1%), C*07:02 (16.2%), and C*01:02 (15.2%) in the current study. In the HLA-DRB1 locus, the three most frequent alleles in Hoa et al. (15), Do et al. (18), and this study were the same, with the top three alleles being DRB1*12:02, DRB1∗09:01, and DRB1*15:02.

The diversity in individual allele frequencies and distributions resulted in differences in frequencies and distributions of HLA haplotypes of combination sets of two, three, or four loci. For instance, the most frequent haplotypes of the HLA-A–B set found in our study were A*11:01–B*15:02 (7.63%) and A*02:01–B*46:01 (5.97%), whereas those sets in Do et al. (18) were A∗29:01–B∗07:05 (6.93%), A∗33:03–B∗58:01 (6.43%), and A∗11:01–B∗15:02 (5.87%); and in Hoa et al. (15) were A*1101–B*15:02 (8.3%) and A*33:03–B*58:01 (5.6%). The existence of A*02:01–B*46:01 in the current study indicates a significant difference. The most frequent haplotypes in the three-locus HLA-A-B-C set by Do et al. (18) were A∗29:01–C∗15:05–B∗07:05, whereas in Hoa et al. (15) and our study were A*11:01–B*15:02–C*08:01. The most frequent haplotype in the three-locus HLA-A–B–DRB1 in Do et al. (18) was A*33:03–B∗58:01–DRB1∗03:01, in Hoa et al. (15) was A*29:01–B*07:05–DRB1*10:01, and in this work was A*11:01–B*15:02–DRB1*12:02.

Many previous studies have reported frequencies of HLA alleles in various populations (19–23). In Korean population (16–18), the most frequent alleles of HLA-A, -B, and -DRB1 were A*02:01, A*02:06, A*11:01, A*24:02, A*31:01, A*33:03 (HLA-A); B*15:01, B*44:03, B*51:01, B*54:01, B*58:01 (HLA-B); and DRB1*01:01, DRB1*04:05, DRB1*07:01, DRB1*08:03, DRB1*09:01, DRB1*13:02, DRB1*15:01 (HLA-DRB1). In the Saudi population, the most common alleles of HLA-A, -B, -C, and -DRB1 are HLA-A∗02:01, A∗24:02 (HLA-A); B∗51:01, B∗50:01 (HLA-B), C∗06:02, C∗07:02 (HLA-C), and DRB1∗07:01, DRB1∗03:01 (HLA-DRB1) (23). Nevertheless, A*24:02, A*33:03, A*02:03 (HLA-A); B*40:01, B*46:01, B*58:01, B*13:01, B*15:02, B*38:02 (HLA-B); C*07:02, C*01:02, C*08:01, C*03:04 (HLA-C); and DRB1*09:01, DRB1*12:02, DRB1*15:01 (HLA-DRB1) were the most common alleles among Han Chinese (22). The most frequent alleles of HLA-A, HLA-B, HLA-C, and HLA-DRB1 in the European American population were A*02:01, A*01:01, and A*03:01 (HLA-A); B*07:02, B*08:01 (HLA-B), C*07:01, and C*07:02 (HLA-C); and DRB1*15:01, DRB1*07:01, and DRB1*03:01 (HLA-DRB1) (25). Furthermore, the most frequent alleles of HLA-A, HLA-B, HLA-C, and HLA-DRB1 in Kinh Vietnamese population found in this study were A*11:01, A*24:02, A*02:01, A*03:03, A*02:03, A*29:01 (HLA-A); B*15:02, B*46:01, B*58:01, B*38:02, B*15:25 (HLA-B); C*08:01, C*07:02, C*01:02, C*03:02, C*15:05, C*03:04 (HLA-C); and DRB1*12:02, DRB1*09:01, DRB1*15:02; DRB1*07:01, DRB1*10:01, DRB1*03:01 (HLA-DRB1).

These data suggest that the distributions and the most frequent HLA alleles in Asian populations, such as Kinh Vietnamese, Han Chinese, and Koreans, were comparable; however, the level of similarity varied from locus to locus. HLA data were collected from a worldwide selection of populations in the Allelefriquencies.net database (http://www.allelefrequencies.net) (26) and used for comparisons. In particular, the distributions of the 10 most frequent alleles of HLA-A, -B, -C, and -DRB1 in Kinh Vietnamese in this study (*n* = 3,750), USA National Marrow Donor Program (NMDP) Vietnamese (*n* = 43,540), South Korean (*n* = 77,584), Japanese (*n* = 24,582), Han Chinese (*n *= 99,672), African American (*n* = 416,581), and European Caucasian (*n* = 1,242,890) were compared and are presented in **Tables 6**–**9**, respectively (26, 27). Although the ranking positions of the 10 most frequent alleles are somewhat different, the distributions of these alleles in the Kinh Vietnamese (observed in this study) and the USA NMDP Vietnamese recorded by ABC are very similar (**Tables 6**–**9**). In particular, eight of 10 HLA-A alleles, nine of 10 HLA-B alleles, 10 of 10 HLA-C alleles, and 10 of 10 HLA-DRB1 alleles among the 10 most frequent alleles in the USA NMDP Vietnamese were the same as those in Kinh Vietnamese. Furthermore, six of 10 HLA-A alleles, four of 10 HLA-B alleles, seven of 10 HLA-C alleles, and four of 10 HLA-DRB1 alleles in the 10 most frequent alleles in USA NMDP South Korea; six of 10 HLA-A alleles, three of 10 HLA-B alleles, six of 10 HLA-C alleles, and six of 10 HLA-DRB1 alleles in the 10 most frequent alleles in USA NMDP Japanese; seven of 10 HLA-A alleles, six of 10 HLA-B alleles, eight of 10 HLA-C alleles, and seven of 10 HLA-DRB1 alleles in the 10 most frequent alleles in USA NMDP Han Chinese; three of 10 HLA-A alleles, two of 10 HLA-B alleles, four of 10 HLA-C alleles, and two of 10 HLA-DRB1 alleles in the 10 most frequent alleles in USA NMDP Africa American; and four of 10 HLA-A alleles, two of 10 HLA-B alleles, five of 10 HLA-C alleles, and three of 10 HLA-DRB1 alleles in 10 highest frequent alleles in USA NMDP European Caucasian are the same as those in Kinh Vietnamese (**Tables 5**–**8**). These data demonstrated that, except for the USA NMDP Vietnamese, among these populations, the similarity of high-frequency HLA alleles in Kinh Vietnamese and Han Chinese was the highest, followed by South Korean, Japanese, European Caucasian, and African American. These data also indicated that the distributions and the most frequent HLA alleles in the Kinh Vietnamese population differed from those in European American and African American populations. These results suggest that Asian populations have different HLA allele frequencies than the American and European populations. Moreover, the diversity in the frequencies of individual alleles of HLA loci creates large differences in frequencies and distributions of two-, three-, or four-loci haplotypes among various populations (19–27). This suggests that transplantations between different populations are quite challenging, and therefore, establishing a bank to store cord blood samples for transplantation programs is important.

**Table 6:** Distribution of the ten most frequent HLA-A alleles in seven populations.

Kinh Vietnamese (in this study) (n = 3750)		USA NMDPSouth Korean* (n = 77584)		USA NMDPJapanese*(n = 24582)		USA NMDPHan Chinese* (n = 99672)		USA NMDP Africa American*(n = 416581)		USA NMDP European Caucasian*(n = 1242890)		USA NMDP Vietnamese* (n = 43540)	
Alleles	Freq.	Alleles	Freq.	Alleles	Freq.	Alleles	Freq.	Alleles	Freq.	Alleles	Freq.	Alleles	Freq.
A*11:01	0.2496	A*24:02	0.2211	A*24:02	0.3530	A*11:01	0.2752	A*02:01	0.1235	A*02:01	0.2755	A*11:01	0.2466
A*24:02	0.1228	A*02:01	0.1857	A*02:01	0.1480	A*24:02	0.1519	A*23:01	0.1099	A*01:01	0.1646	A*33:03	0.1188
A*02:01	0.1122	A*33:03	0.1529	A*11:01	0.0874	A*33:03	0.1011	A*03:01	0.0839	A*03:01	0.1399	A*24:02	0.1109
A*33:03	0.0895	A*11:01	0.1021	A*31:01	0.0849	A*02:07	0.0948	A*30:01	0.0685	A*24:02	0.0846	A*02:03	0.0925
A*02:03	0.0781	A*02:06	0.0747	A*26:01	0.0798	A*02:01	0.0946	A*30:02	0.0667	A*11:01	0.0609	A*02:07	0.0800
A*29:01	0.0703	A*31:01	0.0535	A*02:06	0.0748	A*02:03	0.0774	A*68:02	0.0603	A*32:01	0.0355	A*29:01	0.0753
A*33:01	0.0442	A*26:01	0.0375	A*33:03	0.0648	A*02:06	0.0349	A*74:01	0.0546	A*29:02	0.0353	A*02:06	0.0406
A*02:06	0.0427	A*30:01	0.0317	A*26:03	0.0233	A*30:01	0.0274	A*33:03	0.0518	A*68:01	0.0319	A*02:01	0.0349
A*11:02	0.0315	A*02:07	0.0279	A*02:07	0.0200	A*11:02	0.0263	A*01:01	0.0467	A*26:01	0.0309	A*01:01	0.0332
A*01:01	0.0294	A*01:01	0.0208	A*01:01	0.0100	A*31:01	0.0242	A*02:02	0.0414	A*31:01	0.0270	A*24:07	0.0305
10	0.870	6/10	0.908	6/10	0.946	7/10	0.908	3/10	0.707	4/10	0.886	8/10	0.863

*From the allele frequency net, the grey background indicates common alleles shared between the Kinh Vietnamese and other populations.

**Table 7:** Distribution of the ten most frequent HLA-B alleles in seven populations.

Kinh Vietnamese (in this study) (n = 3750)		USA NMDPSouth Korean* (n = 77584)		USA NMDPJapanese*(n = 24582)		USA NMDPHan Chinese* (n = 99672)		USA NMDP Africa American*(n = 416581)		USA NMDP European Caucasian*(n = 1242890)		USA NMDP Vietnamese* (n = 43540)	
Alleles	Freq.	Alleles	Freq.	Alleles	Freq.	Alleles	Freq.	Alleles	Freq.	Alleles	Freq.	Alleles	Freq.
B*15:02	0.1511	B*51:01	0.0918	B*52:01	0.0990	B*40:01	0.1538	B*53:01	0.1178	B*07:02	0.1306	B*15:02	0.1383
B*46:01	0.1071	B*44:03	0.0850	B*51:01	0.0890	B*46:01	0.1343	B*07:02	0.0729	B*08:01	0.1144	B*46:01	0.1194
B*58:01	0.0765	B*15:01	0.0839	B*35:01	0.0869	B*58:01	0.0874	B*35:01	0.0689	B*44:02	0.0952	B*07:05	0.0798
B*38:02	0.0729	B*58:01	0.0603	B*15:01	0.0769	B*15:02	0.0647	B*15:03	0.0640	B*15:01	0.0606	B*58:01	0.0692
B*07:05	0.0679	B*35:01	0.0599	B*40:02	0.0766	B*13:01	0.0635	B*42:01	0.0531	B*35:01	0.0560	B*38:02	0.0584
B*15:25	0.0555	B*54:01	0.0572	B*44:03	0.0605	B*51:01	0.0457	B*45:01	0.0495	B*40:01	0.0528	B*40:01	0.0533
B*13:01	0.0416	B*46:01	0.0507	B*40:01	0.0603	B*38:02	0.0400	B*44:03	0.0459	B*51:01	0.0473	B*15:25	0.0505
B*40:01	0.0398	B*40:02	0.0467	B*07:02	0.0588	B*54:01	0.0304	B*58:02	0.0422	B*44:03	0.0467	B*13:01	0.0394
B*44:03	0.0326	B*40:01	0.0420	B*40:06	0.0445	B*15:01	0.0300	B*58:01	0.0378	B*18:01	0.0443	B*44:03	0.0275
B*35:05	0.0285	B*48:01	0.0367	B*46:01	0.0410	B*13:02	0.0291	B*08:01	0.0376	B*27:05	0.0373	B*57:01	0.0257
10	0.674	4/10	0.614	3/10	0.694	6/10	0.679	2/10	0.589	2/10	0.685	9/10	0.662

*From the allele frequency net, the grey background indicates common alleles shared between the Kinh Vietnamese and other populations.

**Table 8:** Distribution of the ten most frequent HLA-C alleles in seven populations.

Kinh Vietnamese (in this study) (n = 3750)		USA NMDPSouth Korean* (n = 77584)		USA NMDPJapanese*(n = 24582)		USA NMDPHan Chinese* (n = 99672)		USA NMDP Africa American*(n = 416581)		USA NMDP European Caucasian*(n = 1242890)		USA NMDP Vietnamese* (n = 43540)	
Alleles	Freq.	Alleles	Freq.	Alleles	Freq.	Alleles	Freq.	Alleles	Freq.	Alleles	Freq.	Alleles	Freq.
C*08:01	0.1724	C*01:02	0.1677	C*01:02	0.1732	C*07:02	0.1944	C*04:01	0.2037	C*07:01	0.1600	C*01:02	0.1673
C*07:02	0.1621	C*03:03	0.1195	C*03:03	0.1485	C*01:02	0.1915	C*07:01	0.1170	C*07:02	0.1413	C*08:01	0.1644
C*01:02	0.1521	C*03:04	0.1013	C*03:04	0.1269	C*03:04	0.1165	C*16:01	0.0969	C*04:01	0.1059	C*07:02	0.1553
C*03:02	0.0830	C*14:02	0.0823	C*07:02	0.1218	C*08:01	0.1043	C*02:02	0.0890	C*05:01	0.0939	C*03:04	0.0720
C*15:05	0.0613	C*07:02	0.0822	C*12:02	0.1000	C*03:02	0.0874	C*06:02	0.0865	C*06:02	0.0932	C*03:02	0.0719
C*03:04	0.0561	C*08:01	0.0738	C*14:02	0.0781	C*03:03	0.0473	C*07:02	0.0713	C*03:04	0.0749	C*15:05	0.0718
C*04:03	0.0448	C*03:02	0.0628	C*08:01	0.0682	C*06:02	0.0447	C*17:01	0.0681	C*03:03	0.0534	C*04:03	0.0450
C*04:01	0.0438	C*04:01	0.0556	C*14:03	0.0576	C*04:01	0.0434	C*03:04	0.0565	C*12:03	0.0486	C*06:02	0.0426
C*06:02	0.0410	C*06:02	0.0548	C*04:01	0.0404	C*14:02	0.0406	C*08:02	0.0340	C*02:02	0.0435	C*03:03	0.0397
C*03:03	0.0406	C*14:03	0.0517	C*15:02	0.0196	C*12:02	0.0307	C*05:01	0.0336	C*08:02	0.0385	C*04:01	0.0383
10	0.857	7/10	0.852	6/10	0.934	8/10	0.901	4/10	0.857	5/10	0.853	10/10	0.868

*From the allele frequency net, the grey background indicates common alleles shared between the Kinh Vietnamese and other populations.

**Table 9:** Distribution of the ten most frequent HLA-DRB1 alleles in seven populations.

Kinh Vietnamese (in this study) (n = 3750)		USA NMDPSouth Korean* (n = 77584)		USA NMDPJapanese*(n = 24582)		USA NMDPHan Chinese* (n = 99672)		USA NMDP Africa American*(n = 416581)		USA NMDP European Caucasian*(n = 1242890)		USA NMDP Vietnamese* (n = 43540)	
Alleles	Freq.	Alleles	Freq.	Alleles	Freq.	Alleles	Freq.	Alleles	Freq.	Alleles	Freq.	Alleles	Freq.
DRB1*12:02	0.3096	DRB1*09:01	0.0967	DRB1*04:05	0.1472	DRB1*09:01	0.1554	DRB1*15:03	0.1166	DRB1*15:01	0.1346	DRB1*12:02	0.2838
DRB1*09:01	0.1047	DRB1*04:05	0.0894	DRB1*09:01	0.1387	DRB1*12:02	0.1150	DRB1*07:01	0.1011	DRB1*07:01	0.1342	DRB1*09:01	0.1284
DRB1*15:02	0.0754	DRB1*13:02	0.0862	DRB1*15:02	0.0967	DRB1*15:01	0.1012	DRB1*11:01	0.0854	DRB1*03:01	0.1216	DRB1*10:01	0.0733
DRB1*07:01	0.0668	DRB1*15:01	0.0794	DRB1*15:01	0.0867	DRB1*03:01	0.0681	DRB1*13:02	0.0730	DRB1*04:01	0.0878	DRB1*15:02	0.0709
DRB1*10:01	0.0663	DRB1*08:02	0.0762	DRB1*08:03	0.0744	DRB1*08:03	0.0680	DRB1*03:01	0.0699	DRB1*01:01	0.0860	DRB1*07:01	0.0639
DRB1*03:01	0.0553	DRB1*07:01	0.0715	DRB1*01:01	0.0584	DRB1*11:01	0.0626	DRB1*03:02	0.0631	DRB1*13:01	0.0563	DRB1*03:01	0.0534
DRB1*08:03	0.0450	DRB1*01:01	0.0578	DRB1*13:02	0.0575	DRB1*04:05	0.0612	DRB1*08:04	0.0542	DRB1*11:01	0.0556	DRB1*04:05	0.0485
DRB1*04:05	0.0440	DRB1*12:01	0.0483	DRB1*08:02	0.0434	DRB1*07:01	0.0531	DRB1*13:01	0.0542	DRB1*13:02	0.0488	DRB1*08:03	0.0399
DRB1*15:01	0.0350	DRB1*11:01	0.0473	DRB1*12:01	0.0375	DRB1*16:02	0.0435	DRB1*01:02	0.0392	DRB1*04:04	0.0388	DRB1*15:01	0.0387
DRB1*14:01	0.0261	DRB1*04:06	0.0417	DRB1*14:01	0.0301	DRB1*12:01	0.0342	DRB1*11:02	0.0388	DRB1*11:04	0.0295	DRB1*14:01	0.0304
10	0.823	4/10	0.695	6/10	0.771	7/10	0.763	2/10	0.696	3/10	0.793	10/10	0.831

*From the allele frequency net, the grey background indicates common alleles shared between the Kinh Vietnamese and other populations.

## Institutional Review Board Statement

All participants provided informed consent for inclusion before participating in the study. The study was conducted in accordance with the Declaration of Helsinki, and the protocol was approved by the Ethics Committee of Hanoi Medical University, No. 78/HDDDDHYHN (for DDNCYSH, approval date: 30 May 2017).

## Data Availability Statement

The original contributions presented in the study are included in the article/**Supplementary Material**. Further enquiries can be directed to the corresponding author.

## Author Contributions

TNQ and NDT: hypotheses and research questions. NDT, TNQ,NBK, BQK, and NTVA: research proposal. PDT, NBK, CVS, and TTTA: data analysis. NDT, TNQ, PDT, CVS, and NTVA: Data discussion. NDT, TNQ, TTTA, NTVA, and PDT: manuscript writing. NDT, PDT: Providing figures. NDT, NTVA, BQK,NBK, PDT: final paper review.

## Funding

This study was supported by the National Institute of Haematology and Blood Transfusion in Vietnam and funded by National Research Grant code DTDL.CN-63/19 from the Ministry of Science and Technology, Vietnam.

## Conflict of Interest

The authors declare that the research was conducted in the absence of any commercial or financial relationships that could be construed as a potential conflict of interest.

## Publisher’s Note

All claims expressed in this article are solely those of the authors and do not necessarily represent those of their affiliated organizations, or those of the publisher, the editors and the reviewers. Any product that may be evaluated in this article, or claim that may be made by its manufacturer, is not guaranteed or endorsed by the publisher.
